# Supplementary material for: Complexome profiling of the Chlamydomonas psb28 mutant reveals TEF5 as an early PSII assembly factor
Source: Plant Cell. 2025 Mar 17;37(6):koaf055. doi: 10.1093/plcell/koaf055 (PMC12164590; doi:10.1093/plcell/koaf055)
Supplement: koaf055_Supplementary_Data [file koaf055_supplementary_data.zip › psb28_interactive.zip!/plots/intensity/qualityPlots/peptides/LHL3_Cre03.g199535.t1.1.html]

### Cre03.g199535.t1.1

Trivial name: LHL3  
SeqCoverage [median]: 50.60  
Mass: 27.23 kDa  
  
 

| Sequence | MissedCleavage | Mass [Da] | wtIntensity | muIntensity | Score |
| --- | --- | --- | --- | --- | --- |
| AMATGVTAK | 0 | 848.44 | 4141.13 | 3745.91 | 133.68 |
| AMATGVTAKPVTILQIVDGR | 1 | 2040.14 | 67200.74 | 325067.72 | 220.36 |
| ATAEAPVVEK | 0 | 1013.54 | 636.26 | 0.00 | 94.30 |
| ATAEAPVVEKK | 1 | 1141.63 | 8405.24 | 5086.23 | 128.97 |
| FHLPEAEK | 0 | 969.49 | 10253.05 | 16389.53 | 124.08 |
| FHLPEAEKLNGR | 1 | 1409.74 | 0.00 | 13593.18 | 142.08 |
| FRDDRWIDGR | 2 | 1334.65 | 0.00 | 6543.03 | 63.21 |
| GGTLSGDAAAGK | 0 | 1003.49 | 114452.32 | 123856.44 | 190.02 |
| GGTLSGDAAAGKDAGDK | 1 | 1489.70 | 16629.59 | 12121.47 | 96.67 |
| GGTLSGDAAAGKDAGDKAR | 2 | 1716.84 | 115053.43 | 129905.24 | 148.15 |
| GLIDEATFYDK | 0 | 1270.61 | 33985.36 | 90408.43 | 179.80 |
| KLGPLER | 1 | 811.49 | 77281.56 | 110471.45 | 182.26 |
| PVTILQIVDGR | 0 | 1209.71 | 5809.87 | 0.00 | 210.08 |
| QWNATWDGVR | 0 | 1231.57 | 910.59 | 0.00 | 76.83 |
| RFHLPEAEK | 1 | 1125.59 | 1889.56 | 8719.65 | 97.56 |
| RPSEAADQ | 1 | 872.40 | 32188.90 | 39387.28 | 156.35 |
| TTTDLDK | 0 | 792.39 | 6411.52 | 7687.50 | 134.85 |
| TTTDLDKYK | 1 | 1083.54 | 120676.84 | 106991.26 | 145.17 |
| TTTDLDKYKGLIDEATFYDK | 2 | 2336.14 | 0.00 | 1494.39 | 43.12 |
| WDLSQFK | 0 | 922.45 | 26091.29 | 19709.60 | 95.35 |
|  |  |  | 642017.3 | 1021178.3 |  |
